# Emerging Hybrid Metal Halide Glasses for Sensing and Displays

**DOI:** 10.3390/s24165258

**Published:** 2024-08-14

**Authors:** Wei Tang, Guansheng Xing, Xiuwen Xu, Bing Chen

**Affiliations:** 1 College of Electronic and Optical Engineering and College of Flexible Electronics (Future Technology), Nanjing University of Posts and Telecommunications, Nanjing 210023, China; 2 School of Chemistry and Life Sciences, Nanjing University of Posts and Telecommunications, Nanjing 210023, China

**Keywords:** hybrid metal halides, glass transition, thermodynamics, sensing, imaging

## Abstract

Glassy hybrid metal halides have emerged as promising materials in recent years due to their high structural adjustability and low melting points, offering unique merits that overcome the limitations of their crystalline and polycrystalline counterparts as well as other conventional amorphous semiconductors. This review article comprehensively explores the structural characteristics, electronic properties, and chemical coordination of hybrid metal halides, emphasizing their role in the glass transition from the crystalline phase to the amorphous phase. We examine the intrinsic disorder within the amorphous phase that facilitates light transmission and discuss recent advances in device architecture and interface engineering by optimizing the charge transport of glassy hybrid metal halides for high-quality applications. With full theoretical understanding and rational structural design, potential applications in displays, information storage, X-ray imaging, and sensing are highlighted, underscoring the transformative impact of glassy hybrid metal halides in the fields of materials science and information science.

## 1. Introduction

Organic–inorganic hybrid metal halides (A-B-X) basically consist of continuous or discrete metal halide polyhedrons (B-X), which are segregated by organic cations (A) to form a special spatial configuration [1,2]. The central metal ions (Pb, Sn, Mn, Sb, etc.) with high electron affinity and ionization energy contribute to the formation of the stable polyhedral framework and play a decisive role in the electronic properties of the hybrid metal halide. The organic cations, typically alkylamines or arylamines, serve to stabilize the metal halide polyhedrons and to maintain charge equilibrium. According to the Goldschmidt tolerance factor rule, when the organic cation is overlarge or the metal cation is anisotropic, the typical three-dimensional (3D) stereo configuration will transform into a two-dimensional (2D) layered structure, a one-dimensional (1D) chain structure, or a zero-dimensional (0D) discrete structure [3,4,5].

Due to the strong ionic bonds between the metal and halide ions, the conventional metal halides are ready to form monocrystalline or polycrystalline phases. Monocrystalline metal halides are characterized by a highly ordered structure where atoms, ions, or molecules are arranged in a definitive, repeating pattern extending in all three spatial directions. Polycrystalline metal halides, in contrast, consist of multiple crystalline grains that are randomly oriented and separated by grain boundaries. Polycrystalline metal halides share many properties with their crystalline counterparts but with added complexity due to the presence of tiny grains, since the boundaries can largely influence the material’s electrical, thermal, mechanical, and optical properties [6]. Driven by remarkable physical and photoelectric properties such as high ionic mobility, excellent electrical conductivity, ferroelectricity, and tunable emissions, the crystalline metal halides have been integral to applications including field-effect transistors, lighting, scintillation, and so forth [7,8,9]. For example, the utility of crystalline metal halides in the semiconductor industry stems from their unique electronic properties, which allow for the manipulation of their band gaps and electronic transitions [10,11]. This manipulation is crucial for the development of components such as transistors and diodes. Equally, the importance of crystalline metal halides in X-ray detection and imaging rests on their physical and chemical properties, in particular their capacity for optimizing X-ray absorption, luminescence efficiency, and decay time [12]. These properties are also critical for the enhancement of the performance of detectors and imaging systems in medical imaging, security screening, and material analysis.

Despite the extensive range of applications, monocrystalline and polycrystalline metal halides are sometimes limited in utility owing to their brittleness, processing complexity, or optical scattering. The emergence of glassy metal halides with amorphous structures has addressed the majority of these challenges, opening up new avenues for material design and utility. Unlike their crystalline counterparts, glassy metal halides lack long-range order and grain boundaries, imparting them with unique properties such as structural flexibility and optical transparency [13,14]. The aforementioned properties make glassy metal halides particularly attractive for applications where materials with non-crystalline structures are demanded. For instance, as X-ray scintillators, the inherent disorder within the glassy metal halides causes them to scatter fewer X-rays, resulting in higher transmittance in comparison to polycrystalline scintillators, which is essential for improving the resolution of X-ray imaging [15]. Additionally, the amorphous character of glassy metal halides contributes to good durability and resistance to breakage, thus offering a good lifespan and a reduced maintenance cost [16]. In the field of optoelectronics and photonics, the bendability and processability of glassy metal halides make the lighting layers tightly fitted to the surface of the devices, which is beneficial to applications such as light-emitting diodes (LEDs) and lasers [17]. The specific optical properties of glassy metal halides are being investigated for use in high-resolution imaging and display systems, providing enhanced coloration and resolution over existing technologies. In addition, with the aid of advanced characterization tools including thermal characterizations (e.g., differential thermal analysis (DTA), thermogravimetric analysis (TGA), and differential scanning calorimetry (DSC)), structural characterizations (e.g., variable-temperature X-ray diffraction (VT-XRD), pair distribution function, and extended X-ray absorption fine structure (EXAFS)), optical characterizations (e.g., absorption spectroscopy, infrared spectroscopy, and Raman spectroscopy), and mechanical characterizations (e.g., hardness, and elasticity modulus), researchers have gained deep knowledge about the “structure-property” relation of the glassy metal halides.

This review article delves into the investigation of synthetic strategies employed to produce glassy hybrid metal halides, exploring how different methods impact their properties and applicability. The crystallization kinetics of hybrid metal halides are examined to summarize how the unique structural characteristics affect the glass formation processes. Techniques including melt-quenching, high-pressure compression, and in situ deposition are included to reveal their roles in achieving the desired amorphous structures. On this basis, the review examines how the physical and chemical properties of glassy hybrid metal halides can be tailored through the selection of metal cations and organic cations, as well as their coordination, and concludes the emerging applications of the glassy hybrid metal halides. Through a comprehensive overview of the recent advancements in glassy hybrid metal halides, this review aims to illustrate the pivotal role they play in the fields of materials science and information science.

## 2. Principles of Glass Formation and Manufacturing Techniques

**Figure 1:**
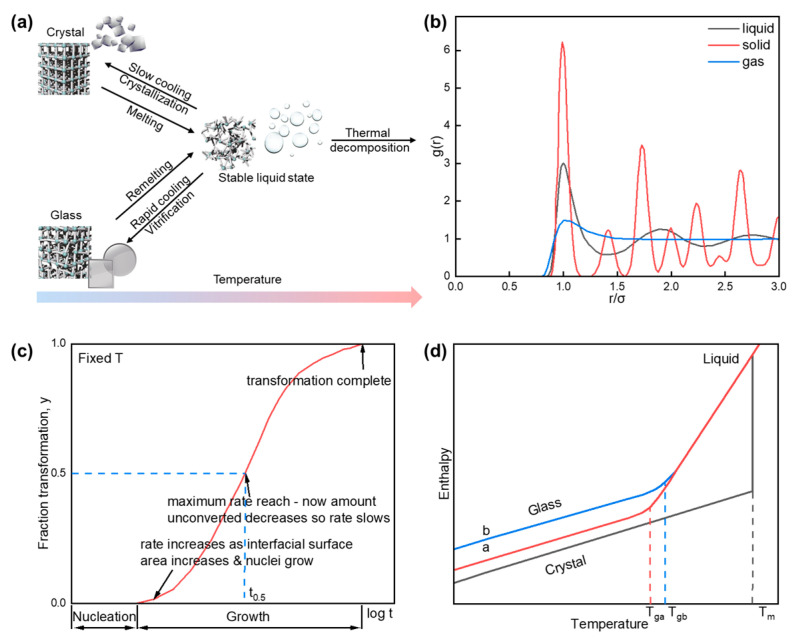
(**a**) Schematic diagram of glass formation. Reproduced with permission from ref. [18]. Copyright 2018, Springer Nature Limited. (**b**) Radial distribution functions for solids, liquids, and glass. (**c**) Diagram of the Avrami equation. Reproduced with permission from Ref [20]. Copyright 2023, The authors, under the terms of the CC-BY 4.0 License. (**d**) The change in enthalpy as a function of temperature for phase transformation at constant pressure. Lines a and b stand for a slow cooling rate and a fast cooling rate for glass formation, respectively. Reproduced with permission from ref. [21]. Copyright 2001, Springer Nature Limited.

Following the standard set forth by the American Society for Testing and Materials (ASTM) [15], glass is an inorganic product of fusion that has cooled to a rigid condition without crystallizing. This definition was once used to distinguish glass from other materials like ceramics or polymers, which are also rigid or non-crystalline, respectively, but do not share the same formation process. In a broad sense, glasses can be described as amorphous solids (inorganics, organics, metals, and organic–inorganic hybrids) with the phenomenon of vitrification, the definition of which is the transformation of a material into an intermediate phase during cooling to a solid with distinct physical properties (Figure 1a) [14,18]. Inorganic glass is an amorphous solid material that is typically composed of inorganic oxides, fluorides, silicates, phosphates, borates, etc. In contrast, organic glass, also known as organic polymer glass, refers to a type of glass-like material that is composed of organic compounds, typically polymers (e.g., polymethyl methacrylate). Organic glass is generally light, flexible, and impact-resistant. Metallic glass, also known as amorphous metal, is a type of material that exhibits a disordered atomic structure similar to that of traditional glass but is composed of metallic elements or alloys. The metallic glasses have glass-like properties such as high strength, hardness, superior magnetic properties, corrosion resistance, and deformation resistance. Organic–inorganic hybrid glass is a type of material that combines both organic and inorganic components in a single, integrated structure. The typical examples are metal-organic framework glasses and organic–inorganic hybrid halide glasses. The absence of long-range ordered crystalline structure in the amorphous phase can be evidenced by the radial distribution function that is directly linked to the number of metal coordination sites (Figure 1b) [19].

For glass formation, four specific temperatures are important, viz., glass transition temperature (*T_g_*), crystallization temperature (*T_x_*), melting temperature (*T_m_*), and decomposition temperature (*T_d_*). The definition of *T_g_* is the temperature at which a material changes from solid to glass. *T_m_* and *T_x_* are the specific temperatures at which a substance changes from a solid state to a liquid state and from a liquid to a solid (an ordered crystal), respectively. *T_d_* is the temperature at which a substance begins to break down or undergo a chemical change, losing its original properties. From the thermodynamics point of view, glass formation should meet the key conditions between *T_g_*, *T_x_*, *T_m,_* and *T_d_*, viz., *T_g_* < *T_x_* < *T_m_* < *T_d_* [22,23,24]. For example, the *T_g_*_,_ *T_x_*, *T_m,_* and *T_d_* of the [(*S*)-(−)-1-(1-naphthyl)ethylammonium]_2_PbBr_4_ glass have been reported at 67.2 °C, 101.1 °C, 173.1 °C, and 205.0 °C, respectively [23]. Importantly, the glasses need to have the ability to mobilize and recrystallize components during the heating process. The *T_m_* of the glass should be lower than its *T_d_* to ensure that decomposition does not occur during heating. When the temperature of the liquid decreases from *T_m_*, the liquid shear viscosity (*ƞ*) gradually increases. The change of viscosity as a function of temperature (*T*) is typically described using the Vogel–Fulcher–Tammann equation [25]:

(1) $$\eta =\eta_{0}exp\left[\frac{a}{T-T_{0}}\right]$$

where $\eta_{0}$ and a are empirical material-dependent constants, and *T*_0_ is an empirical fitting parameter (typically lies about 50 °C below the glass transition temperature). Specifically, at a given temperature range, when the viscosity reaches the solid–liquid critical viscosity (typically ~10^14^ N s m^−2^), the *ƞ* value will increase significantly, and the flow of the liquid becomes more difficult due to stronger molecule–molecule or ion–ion interactions, contributing to the transformation of the liquid into a glassy phase [26].

From the kinetics point of view, homogeneous nucleation is ready to form in the liquid at the same time when the temperature drops [27]. The Avrami equation, which describes the process of phase transformation of both glass and crystal at constant temperature, has been used to study the crystallization kinetics of glass [28]:

(2) $$x\left(t\right)=1-exp\left[-{\left(Kt\right)}^{n}\right]$$

where *x* is the crystalline volume fraction, *t* is a moment in time, *n* is the Avrami constant, and *K* is a reaction constant related to the activation energy and temperature. From the above equation, it is easy to find that *n* and *E* are the key parameters for evaluating the kinetics of glass crystallization. For example, Mitzi and coworkers studied *S*-NPB glasses to investigate the kinetic effects of the crystal-glass transition [23,29]. The *E* (334 ± 21 and 365 ± 25 kJ mol^−1^) and *n* (2.02 ± 0.11) values were obtained for the first time under non-isothermal conditions by combining the two dynamic models of Ligero [30] and Kissinger [31], which made great contributions to the subsequent evaluation of the glass formation ability and stability of different systems. The initial slow growth rate in the early state is attributed to the time required for the formation of a significant number of nuclei in the new phase, as shown in Figure 1c. In the intermediate period, the transformation from nuclei to particles is quite fast, during which the nuclei continue to form in the remaining parent phase. As deduced from the Avrami equation, it is evident that the rate of cooling is of paramount importance in the successful preparation of glassy phases. If the rate of cooling is relatively slow, the liquid molecules or ions have sufficient time to rearrange themselves and grow in an orderly fashion on the surface of the nucleus, therefore forming a long-range, ordered crystalline structure. On the contrary, if the cooling rate is fast enough (typically ~6000 °C s^−1^) [32], the liquid molecules’ crystallization rate is low with a large degree of subcooling to form a “subcooled liquid” glassy phase. Consequently, different cooling rates result in the enthalpy of both the glass and the crystal being shifted away from the liquid line (Figure 1d). The enthalpy of the glass formed under faster cooling rate conditions is higher than the enthalpy of the liquid, and the structure is of lower energy; the enthalpy of the crystal formed under slower cooling rate conditions is lower than the enthalpy of the liquid, and the structure is of lower energy. In this regard, glass can be deemed a biphasic mixture of highly ordered and highly disordered materials, with an ordered arrangement of tiny grains measuring approximately 3 or 4 molecular diameters [26]. Previous reports by David Turnbull have indicated that the potential for glass formation follows an empirical relationship known as the Turnbull criterion [26,33,34], as below:

(3) $$\frac{T_{g}}{T_{m}}\;\geq \;0.67$$

When the *T_g_*/*T_m_* value is within the range of the Turnbull criterion, it indicates a strong ability to form glass. To facilitate such a fast cooling rate, the temperature difference between the *T_g_* and *T_m_* should not be too large.

For the material to form a glassy phase, the large intermolecular space is conducive to preventing the formation of crystal structures during quenching [35]. In this regard, organic–inorganic hybrid metal halides with alkyl chains are good candidates for glasses, owing to their high flexibility for structural transformations and conformational changes below their melting [36,37,38]. In addition, metal ions with smaller coordination sites are beneficial for glass formation [26,39]. The lower the coordination number, the lower the number of hydrogen bonds of the type A–H⋯X (A is N, P, O, etc., and X is halogen), ultimately leading to a lower *T_m_* [40]. Additionally, the electronegativity of the halide ions and the size of the radius of the metal ions are also key factors affecting the *T_m_.* The selection of a large volume of halogen ions or a small volume of metal ions is a common method to reduce the *T_m_* [38,41].

**Figure 2:**
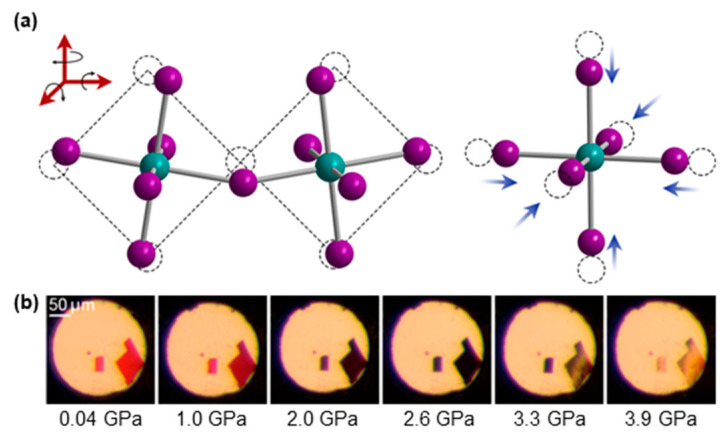
(**a**) The effect of high pressure on the structure of metal halides. Green and purple spheres represent metals and halides, respectively. Dashed lines indicate the original atom positions. Reproduced with permission from ref. [48]. Copyright 2017, American Chemical Society. (**b**) Piezochromism in single crystals of (MA)_2_[PbI_2_(SCN)_2_]. Reproduced with permission from ref. [42]. Copyright 2016, American Chemical Society.

In addition to conventional rapid cooling, hybrid metal halide glasses can also be prepared by high-pressure compression [42]. Unlike traditional glass-making techniques that rely on melting, this method uses high-pressure compression to consolidate the powdered precursors into a dense form at room temperature or slightly elevated temperatures. Specifically, high pressure induces contraction of the B–X bonds as well as distortion of the lattice [43,44,45], resulting in a phase transition to form a disordered amorphous phase glass (Figure 2a) [46]. Such contraction and distortion adjust the orbital interactions of metal halides [45,47], and therefore change the thermodynamic distribution of the lattice [48]. After compression, the material may undergo a sintering process at elevated temperatures in controlled atmospheres to further densify the material and enhance its properties. The sintering process helps in achieving better bonding between particles and can remove any remaining porosity. The glass is then cooled under controlled conditions to retain its amorphous structure. In some cases, an annealing step may follow to relieve internal stresses and improve the mechanical stability and optical clarity of the glass. For 2D hybrid metal halides, high pressure can cause a spatial alteration of the layer spacing, which significantly affects the dielectric properties [49,50]. For example, piezochromism has been reported in (MA)_2_[PbI_2_(SCN)_2_] crystals, which show a phase transition at 2.3 GPa and a glass transition at around 3.8 GPa (Figure 2b) [42]. With the increase in pressure, the crystal of 2D (MA)_2_[PbI_2_(SCN)_2_] not only shrinks in different degrees along the *a*, *b*, and *c* axes, but also the contraction of the layer spacing becomes more obvious. When the pressure increases from 0 to 3.9 GPa, the *a*, *b*, and *c* axes are compressed by 7.3%, 5%, and 6%, respectively. In addition, the layer spacing is decreased by 7.4%, which affects the electronic structure and orbital interactions. Correspondingly, the band gap gradually decreases from 2.14 eV to 1.8 eV, with the color changing from translucent red to opaque black and finally translucent yellow. The high-pressure compression technique allows for the exploration of new glass systems that are difficult or impossible to create through traditional melting processes due to the high volatility or decomposition of components at elevated temperatures. The ability to process materials at lower temperatures also opens up possibilities for incorporating sensitive materials that might degrade or lose functionality under high heat.

**Figure 3:**
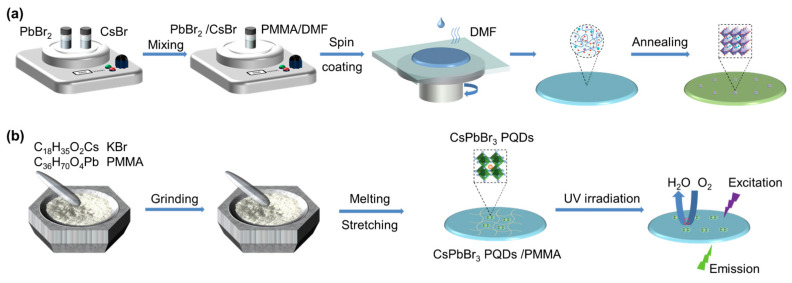
(**a**) Schematic of in situ deposition of CsPbBr_3_-based polymer glass. Reproduced with permission from ref. [55]. Copyright 2021, Wiley-VCH GmbH. (**b**) Schematic of in situ deposition of polymer fusion encapsulation. Reproduced with permission from ref. [56]. Copyright 2021, Wiley-VCH GmbH.

Apart from melting, rapid cooling, and high-pressure compression, generalized hybrid metal halide glasses can also be prepared by in situ deposition, which induces the capsulation of nanosized hybrid metal halides in the second matrix, such as silicate glasses and polymers [51,52]. The metal-halide precursors react or decompose to form nanosized particles. The conditions under which this occurs—such as temperature, pressure, and the chemical environment—are crucial for controlling the size, distribution, and stability of the metal halides. Once the metal halide particles are formed and stabilized within the matrix, the composite material is cured or solidified. For example, after dissolving CsBr and PbBr_2_ in a DMF solution, CsPbBr_3_–polymer glass can be obtained by adding the precursor solution to a polymer (polymethylmethacrylate or polyvinylidene fluoride) solution (Figure 3a) [53,54]. Finally, during the spin-coating and fading processes, the polymerization of the polymer itself and the strong coordination reaction between the oxygen atom and Pb promote the in situ nucleation of nanocrystals in the amorphous phase with high transparency [55].

Except for solution-based polymerization, an alternative approach is to use an in situ melt encapsulation technique that is free of organic solvents to avoid solvent degradation of the luminescent properties of the glass (Figure 3b) [56,57]. Through sufficient stirring and melting operations, the polymer matrix and metal halide precursors are fully mixed to promote the in situ formation and dispersed growth of metal halide quantum dots, and ultimately, various shapes of glass products are prepared under the action of external forces. The hybrid metal halide glasses obtained in this way are effectively shielded from the bursting effect of water and oxygen on the optoelectronic properties of the glass. For silicate glasses, the in situ melt encapsulation technique might involve a heat treatment to finalize the glass formation. In situ deposition provides a controlled way to integrate functional nanomaterials into stable and usable forms and offers a unique approach to modifying the structural and functional properties of the resulting composite materials. Despite the advantages, however, the melt rapid cooling process offers better controllability and stability in comparison to both high-pressure compression and in situ deposition approaches, resulting in the preparation of hybrid glass films with higher quality and properties. Therefore, the preparation of hybrid metal halides refers in particular to the rapid cooling of the melt throughout the subsequent paragraphs, unless otherwise stated.

## 3. Glassy Hybrid Metal Halides and Derivates

Hybrid metal halide glasses can be classified based on the type of central metal ions such as Pb, Sn, Mn, and Sb present in their structure. These central metal ions significantly alter the coordination with halide ions and influence their processing, functionality, and suitability for various advanced technological applications of the glasses. Additionally, by using the special processing methods, crystalline–amorphous heterojunction can be harvested, which expands the research scope of hybrid metal halides.

### 3.1. Pb-Based Hybrid Metal Halide Glasses

Due to the volatile and destructible nature of the organic cations at high temperatures, the majority of hybrid lead halides will decompose before melting during the heat process. Therefore, it is necessary to study the relationship between *T_m_* and *T_d_* and the way of reducing *T_m_* before preparing the glasses (Table 1) [58]. By adjusting the types and ratios of the organic cations, it is possible to achieve a precise regulation of the properties of 2D lead halide glasses, including the optimization of the range of light absorption, electrical conductivity, and thermal stability [59,60]. As a special kind of Pb-based hybrid metal halide, Pb-based hybrid organic–inorganic perovskites (HOIPs) have shown great promise with tailored thermodynamic and optoelectronic properties. Recent reports have shown that modification strategies such as *N*-methylation or I-substitution of organic cations can limit the rotation and vibration of organic components, which can effectively reduce the *T_m_* of HOIPs [61,62]. Especially for the I-substitution strategy, the additional I–I interaction can improve the viscosity of the molten liquid and enhance the stability of the molten liquid at the same time, which makes the octahedral dimer of perovskite arrange in a wave-like layer [62], conducive to the reversible switching of HOIPs between glass and crystal.

**Figure 4:**
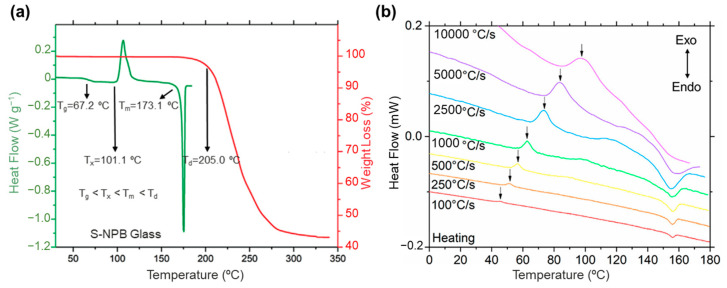
(**a**) DSC (green line) and TGA (red line) of the *S*-NPB glass. Reproduced with permission from ref. [22] Copyright 2020, Wiley-VCH GmbH. (**b**) kinetic effects on the crystallization of 1-MeHa_2_PbI_4_ glass. Reproduced with permission from ref. [32]. Copyright 2023, American Chemical Society.

As a typical example, Mitzi and coworkers reported the first study on the preparation of a 2D [(*S*)-(−)-1-(1-naphthyl)ethylammonium]_2_PbBr_4_ glass (termed *S*-NPB glass) with a large tendency to form glass [22]. In-depth research indicated that the larger chiral molecule played a crucial role in enlarging the lattice volume and facilitating the formation of short-range ordered glass structures during the melt-cooling process. It should be mentioned that hydrogen bonding interactions between the bromine atoms and the organic cations play an important role in facilitating the glass formation of organic molecules in the particular configuration of a parallel arrangement. Due to its unique structural characteristics, the *S*-NPB glass can form even at slower cooling rates (e.g., 20 °C min^−1^) compared to conventional fast cooling methods. The DSC and TGA of the *S*-NPB glass showed that the melt-quenched glassy *S*-NPB reveals clear transition temperatures of *T_g_* ~67 °C, *T*_x_ ~101 °C, *T_m_* ~173 °C and *T_d_* ~205 °C, respectively (Figure 4a). The *S*-NPB glass showed a *T_g_*/*T_m_* value of 0.76, which falls in the range of the Turnbull glass-forming ability criterium (*T_g_*/*T_m_* > 0.67), indicating a strong propensity toward glass formation and supporting such a small critical cooling rate. In a recent report, by using flash DSC, the authors extended the range of metal halide glass formation across a broader range of organic (fused ring to branched aliphatic) and halide (bromide to iodide) compositions. For example, 1-MeHa_2_PbI_4_ (1-MeHa = 1-methylhexylammonium) has a broader range of compositions and crystallization kinetics, spanning from 100 to 10,000 °C s^−1^ (Figure 4b) [32]. The calculated *T_g_*/*T_m_* value for 1-MeHa_2_PbI_4_ is 0.66, which is smaller than the Turnbull criterium value. The great glass formation ability is probably attributed to the strong flexibility of the 1-MeHa cation in contrast to the rigid chiral molecules in *S*-NPB, as mentioned before.

**Figure 5:**
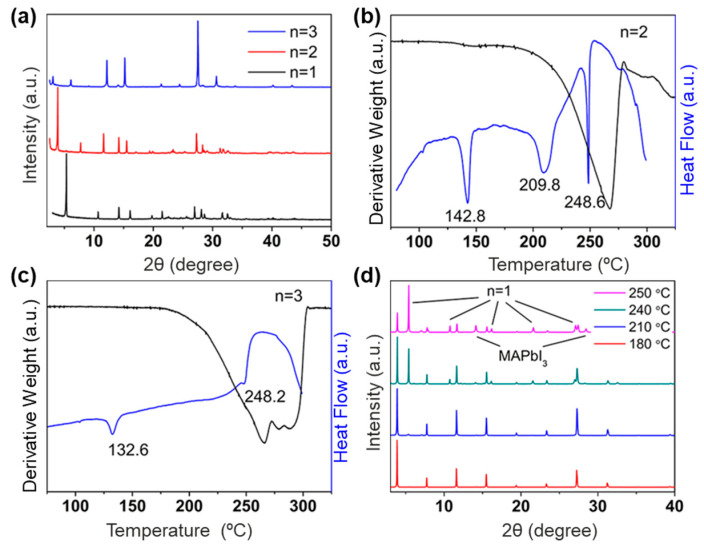
(**a**) X-ray diffraction (XRD) patterns of metal halide perovskites with *n* = 1, 2, and 3, respectively. (**b**,**c**) TGA and DSC curves of metal halide perovskites with *n* = 2 and 3, respectively. (**d**) Non-in situ XRD patterns of layered metal halide powders (*n* = 2, annealed at less than 200 °C for 30 s). Reproduced with permission from ref. [65]. Copyright 2019, American Chemical Society.

Except for organic cations, the thickness of the inorganic layer, namely the *n* value, also largely affects the thermodynamic properties of lead halide perovskites. This is attributed to alterations in the strength of hydrogen bonding interactions between the organic and inorganic components, as well as other microstructural changes within the inorganic layer. Furthermore, the results indicated that the melt-treated films prepared under ambient conditions exhibited a high degree of phase purity and crystallinity, which provided valuable references and insights for future related studies, particularly for *n* = 1. However, during the melting process, Pb-based metal halides containing *β*-methylphenylethylammonium failed to co-melt when the value of *n* was 2 or 3, because phase separation occurred at the point of inclusion crystals, resulting in mixtures of both 2D and 3D impurities (Figure 5) [65]. From their thermodynamic spectra, it can be found that these 2D lead halides with different layers remain stable at 200 °C and transform the structures below 200 °C. In particular, the diffraction peak of the lead halide with an *n* value of 2 becomes sharper with the increase in temperature, which indicates that its crystallinity gradually increases. When the temperature rises to 210 °C, the hybrid lead halide with an *n* value of 1 begins to melt, and a large amount of melted hybrid lead halide with an *n* value of 1 is produced at 250 °C, so that the final mixture of hybrid lead halide with *n* = 1, 2, and 3D MAPbI_3_ is obtained. In addition, the tunability of the organic cations of 2D hybrid lead halides allows for further tuning of the glass composition through cation exchange in either the liquid phase or the solid phase [64,66,67,68]. Although significant progress has been made in the field related to hybrid lead halide glass with excellent performance [69,70,71], the issue of lead toxicity persists as a vital problem [72].

### 3.2. Sn-Based Hybrid Metal Halide Glasses

This issue of Pb toxicity can be effectively addressed by replacing Pb^2+^ with a more environmentally friendly Sn^2+^ ion [73]. As an added benefit, for 2D Sn-based hybrid metal halides possessing the same organic cation as Pb-based hybrid metal halides, the *T_m_* is typically 25~45 °C lower, probably due to the higher bond ionicity of their Sn-based inorganic backbone [66,74,75].

By introducing several related phenylethylammonium cations into tin iodide, Mitzi and coworkers first prepared the 2D heterogeneous tin iodide films on flexible plastic substrates and observed a change in the specific hydrogen bonding between the organic cation and the inorganic component during the melting process [66]. This change led to a minor structural adjustment of the inorganic framework while lowering the melting temperature of the material (*T_m_* ≤ 200 °C) to well below its decomposition temperature. This research result provides a new research idea and development direction for the preparation of more stable and better-performance tin iodide-based metal halide materials. Subsequently, the same group reported a series of structurally similar hybrid tin iodides (R-NH_3_)_2_SnI_4_ (the R-NH_3_^+^ cation is derived from the substituted phenethylamine) [74]. Although the primary unit of the organic cation remains the phenethylammonium cation, a variety of atoms or groups (e.g., halogens, methyl groups) are chosen to modify the phenethylammonium cation at different positions in contrast to the above. The subtle differences between different atoms or groups (e.g., small differences in van der Waals forces) affect the stacking mode of the phenethylammonium cation and cause subtle distortions in the stereo-structure of the tin iodides, which is of great importance for the alteration of the thermodynamic parameters of the hybrid tin iodides [76]. The experimental results reveal that the halogen in the benzene ring with *para*-, *meta*-, and *ortho*-substitution positions undergoes a gradual change in *T_m_*. The greater the electronegativity of the halogen atom, the lower the *T_m_*. In particular, chiral cations containing racemic substituents exhibit the lowest *T_m_* and phase transition temperatures [77].

Although Sn-based hybrid metal halide glasses are gaining attention in photovoltaic and optoelectronic applications due to their favorable electronic properties and lower toxicity compared to lead-based glasses, there is a potential problem associated with these materials, namely, the stability issue. This instability of Sn^2+^ can manifest as degradation when exposed to environmental factors like moisture, oxygen, and light. The degradation process is accelerated due to the oxidation of Sn^2+^ to Sn^4+^, making the fabrication process more complex (such as in inert gas atmospheres or vacuum conditions).

### 3.3. Mn-Based Hybrid Metal Halide Glasses

In the search for friendly metal halides, Mn-based metal halide glasses represent a fascinating candidate. The Mn^2+^ ion, with its d^5^ electron configuration, is known for its strong and stable luminescence, which can be tuned by varying the host matrix and the halide environment [78]. Due to the distinctive light absorption and emission characteristics of Mn^2+^, these Mn-based hybrid metal halide glasses have significant potential for advanced applications. In addition, a variety of Mn-based hybrid metal-halides have been reported with low *T_g_* and *T_m_*, suitable for the preparation of optical glasses (Table 2). The unique photophysical properties of Mn^2+^ allow these glasses to serve in roles ranging from laser technology to lighting and display technologies.

**Figure 6:**
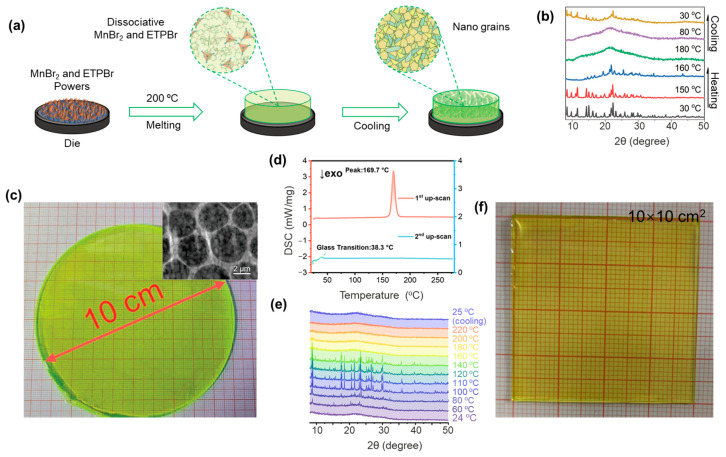
(**a**) The scheme for fabricating (ETP)_2_MnBr_4_ transparent medium. (**b**) Variable temperature XRD of the glass formation process of (ETP)_2_MnBr_4_. (**c**) An image of (ETP)_2_MnBr_4_ (10 cm in diameter) prepared by the casting method. Inset: cryogenic transmission electron microscopy image of the glass formed by (ETP)_2_MnBr_4_ Reproduced with permission from ref. [81]. Copyright 2022, Wiley-VCH GmbH. (**d**) Thermal analysis of crystalline (HTPP)_2_MnBr_4_. (**e**) Variable temperature XRD of (HTPP)_2_MnBr_4_ glass. (**f**) Photographs of glassy (HTPP)_2_MnBr_4_ under visible light. Reproduced with permission from ref. [15]. Copyright 2022, Wiley-VCH GmbH.

In a typical study of Mn-based hybrid metal halide glasses, Xia and coworkers reported the preparation of (ETP)_2_MnBr_4_ (ETP = thyltriphenylphosphonium) glass by direct heating of ETPBr and MnBr_2_ powders to 200 °C to melt and subsequently cooling to room temperature using a graphite mold (Figure 6a) [81]. Note that *T_g_*/*T_m_* for (ETP)_2_MnBr_4_ is significantly larger than the empirical value of 0.67, showing the strong glass formation ability. Variable temperature-dependent XRD patterns of (ETP)_2_MnBr_4_ transparent glass indicated that the diffraction peaks of (ETP)_2_MnBr_4_ crystals gradually became weak and completely disappeared as the temperature increased from 30 to 180 °C, and (ETP)_2_MnBr_4_ crystals will be observed again when slowly cooling back to 30 °C (Figure 6b). This experimental result demonstrated that although characteristic reflections of the amorphous phase appeared in the XRD pattern of the (ETP)_2_MnBr_4_-based transparent medium related to the formation of the amorphous phase and the as-prepared (ETP)_2_MnBr_4_ glass are transparent through rapid cooling, the occurrence of recrystallization still happens (Figure 6c).

In another example, Kuang and coworkers reported the preparation and optical properties of (HTPP)_2_MnBr_4_ (HTPP = hexyltriphenylphosphonium) glasses for X-ray imaging [15]. The authors selected HTPP as the cation because it exhibited high spatial site resistance, which was beneficial for constructing Mn-based glasses. Thermal analysis revealed that the *T_g_* and *T_m_* of such crystals were 38.3 and 169.7 °C (Figure 6d), which were substantially lower than their *T_d_* (~280 °C). Yet, the ratio of *T_g_*/*T_m_* is consistent with the empirical value for glass formation. Temperature-dependent XRD patterns showed that the glassy samples exhibit a diffuse diffraction pattern at room temperature, and the crystallinity increases upon further increasing the temperature (Figure 6e). When the temperature reaches 110 °C, the XRD pattern is in line with the (HTPP)_2_MnBr_4_ crystals. When the temperature was further elevated to 160 °C (or above) and quenched, the glassy (HTPP)_2_MnBr_4_ could be obtained. Through a low-temperature melt-quenching process, the authors obtained an impressive large-area transparent luminescent (HTPP)_2_MnBr_4_ glass with 28.5% PLQY and >78% transmittance in the range of 506–800 nm (Figure 6f), which had potential for use in X-ray imaging screens, solid-state lighting, and various optical applications.

It is worth noting that for the preparation and recycling of Mn-Br glass systems, the instability of manganese bromide is a big issue since it is susceptible to decomposition and deliquescence at elevated temperatures [40]. In this regard, Jin and coworkers developed a 0D hybrid manganese halide glass with low *T_m_* (175 °C), namely BTA_2_MnBr_4_ (BTA = benzyltrimethylammonium), by optimizing the melting process [80]. To implement this, a certain amount of hybrid manganese halide powder was first placed on a quartz wafer and heated to *T_m_* for melting. Thereafter, the preheated quartz wafer was used to gently press the molten material to flatten the surface before the melt cooled down. This meticulous process will help to improve the quality of the glass and provide a better basis for subsequent applications. In the preparation of thin-film transistors and semiconductor transistors, this glass protects the electrodes with their pixelated structure from damage at higher temperatures (>200 °C). Except for the unique structural and optoelectronic properties, by replacing the A-site organic cations, the hybrid manganese halides can also enhance the exciton binding energy, modulate the ion migration activation energy [85,86,87], improve carrier transport capacity, and reduce the dark current [88]. Therefore, the hybrid manganese halides are believed to have a wide range of applications, not only for scintillation but also for direct X-ray imaging.

### 3.4. Sb-Based Hybrid Metal Halide Glasses

Due to the greater ionic attraction of Sb^3+^ ions compared to divalent Pb, Sn, and Mn ions in pure inorganic metal halides, it is expected to lower the melting point and prepare glasses by melt processing for Sb-based hybrid metal halides (Table 3) [89]. This effect is rooted in the modulation of supramolecular interactions between the organic components as well as hydrogen-bonding interactions between the organic and inorganic components in Sb-based hybrid metal halide systems.

**Figure 7:**
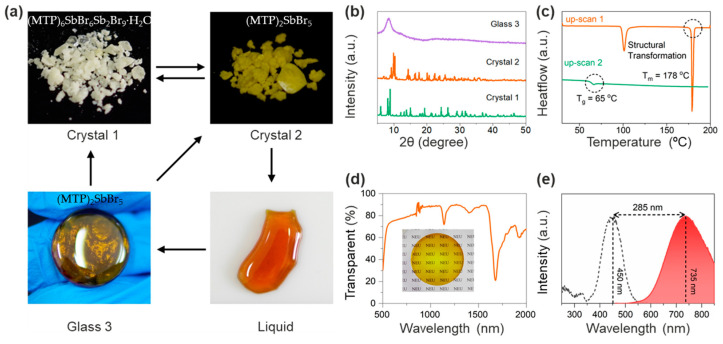
(**a**) Phase transitions of the (MTP)_6_SbBr_6_Sb_2_Br_9_·H_2_O crystal (crystal 1), (MTP)_2_SbBr_5_ crystal (crystal 2), and the melt and amorphous counterpart (glass 3), respectively. (**b**) XRD of crystal 1, crystal 2, and glass 3, respectively. (**c**) DSC of crystal 1. (**d**) The transmission spectrum of glass 3. (**e**) PLE and PL spectra of glass 3. Reproduced with permission from ref. [90]. Copyright 2022, Wiley-VCH GmbH.

The first example of Sb-based hybrid metal halide glass, (MTP)_2_SbBr_5_ (MTP = Methyltriphenylphosphonium), was reported by Xu and coworkers in 2022 [90]. The as-prepared (MTP)_2_SbBr_5_ crystals can be melted at 190 °C, and their amorphous counterparts can be obtained by directly cooling them down to room temperature without the need for rapid cooling (Figure 7a,b). Interestingly, the (MTP)_2_SbBr_5_ has a reversible structural phase transition toward its hydrous form, (MTP)_6_SbBr_6_Sb_2_Br_9_·H_2_O. The anionic part of the (MTP)_6_SbBr_6_Sb_2_Br_9_·H_2_O crystal consists of octahedral [SbBr_6_]^3−^ and dimeric [Sb_2_Br_9_]^3−^, whereas the anionic part of the (MTP)_2_SbBr_5_ crystal consists of [SbBr_5_]^2−^ only. The amorphous liquid was obtained when the as-prepared crystal was further heated beyond 190 °C (*T_m_* ~178 °C). The (MTP)_2_SbBr_5_ glass phase can be obtained when the temperature decreases, which can be sequentially recrystallized into (MTP)_2_SbBr_5_ crystal and (MTP)_6_SbBr_6_Sb_2_Br_9_·H_2_O crystal by triggering with acetonitrile and H_2_O vapor, respectively. From the DSC curve, it can be deduced that the structural transformation from (MTP)_6_SbBr_6_Sb_2_Br_9_·H_2_O to (MTP)_2_SbBr_5_ occurs at ~100 °C (Figure 7c). The (MTP)_2_SbBr_5_ glass exhibits high transparency (>75%) and excellent luminescent properties excited by blue light and is able to emit light in the near-infrared region, coinciding as the first case of a Sb-based hybrid metal halide glass material with blue-light excitation and NIR emission characters (Figure 7d,e). The large Stokes shift is attributed to the significant deformation of the structure due to the vibration of the Sb-Br bond in (MTP)_2_SbBr_5_ glass.

**Figure 8:**
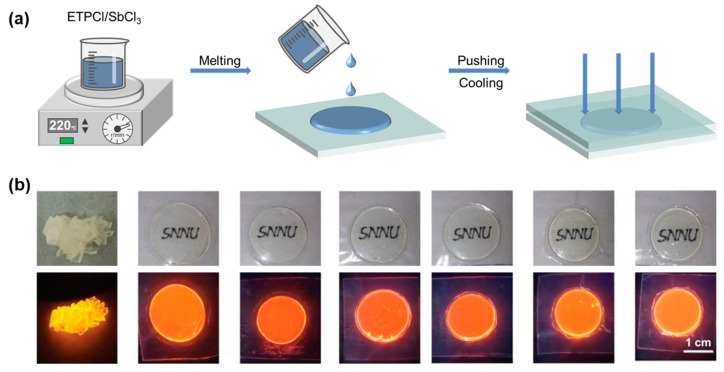
(**a**) Scheme of the synthesis process of the (ETP)_2_SbCl_5_ wafer. (**b**) Photographs of (ETP)_2_SbCl_5_ crystals and glass wafers. Reproduced with permission from ref. [91]. Copyright 2023, Wiley-VCH GmbH.

Inspired by the large Stokes shift in Sb-based hybrid metal halide, Liu and coworkers reported the preparation of (ETP)_2_SbCl_5_ glass wafers for large-sized transparent scintillators with a high photoluminescent quantum yield and self-absorption-free properties in a follow-up study [91]. The target (ETP)_2_SbCl_5_ glass was obtained by melting a mixture of ETPCl and SbCl_3_ at 220 °C and rapidly cooling the melt on stainless steel plates to room temperature (Figure 8a). The (ETP)_2_SbCl_5_ glass wafers with *T_g_*/*T_m_* conformed to the Turnbull criterium, demonstrating good glass formation ability. It should be mentioned that in this setup, the thickness of the soon-to-be-formed glass wafer can be fine-adjusted by squeezing, which is important to control the balanced properties such as transparency and absorbance (Figure 8b). In another example, Zhang and coworkers reported Sb-based metal halide glasses containing bulky chiral cations, all of which satisfy the following thermodynamic parameters: *T_g_* < *T_x_* < *T_m_* < *T_d_* and *T_g_*/*T_m_* > 0.67 [24]. The presence of abundant hydrogen-bonding interactions in (*R*/*S*-2-HMM)_3_SbCl_6_ (2-HMM = 2-(hydroxymethyl)morpholine) increases the melt viscosity during melt cooling, thus avoiding the generation of long-range ordered crystals. Therefore, 0D (*R*/*S*-2-HMM)_3_SbCl_6_ crystals can easily form glassy phases by melt quenching with the ability of reversible phase transformation. Combining with the Kissinger model yields an *E*-value as low as 105 ± 8 kJ mol^−1^, which is much lower than that reported by Singh for S-NPB [23]. The glass phase exhibits good transparency in the 400−800 nm range. More importantly, because of the radiative recombination of self-trapped excitons due to [SbCl_6_]^3−^ lattice distortions, the (*S*-2-HMM)_3_SbCl_6_ crystals and (*S*-2-HMM)_3_SbCl_6_ glasses exhibit bright yellow and orange emissions, respectively. Note that the non-chiral (*S*-2-HMM)_3_SbCl_6_ crystals can also melt to form glass, but the difference between their crystal and glass phase photophysical characteristics is small [93].

Similar to most hybrid metal halides, the instability of Sb-based hybrid materials often occurs during the melting process, resulting in the inability of the melt to recrystallize and reorder during cooling. To circumvent these issues, In a recent study, Mitzi and coworkers prepared (*S*-MeTMPZ)SbI_5_ (*S*-MeTMPZ = (*S*)-1,1,2,4,4-pentamethylpiperazinium) glasses by initially transferring the preheated samples to a special hot press and promptly quenching them under a specific pressure after a mere 5 s at a high temperature (*T_m_* = 252.4 °C) [89]. The authors demonstrated that Sb-analogues melt at lower temperatures in comparison to other metal halides. This observation was attributed to the structural changes induced by the increased stereochemical activity of the Sb^3+^ lone pair, which was coupled with the reduction in effective dimensionality due to steric interactions with the organic cations. The lower melting temperatures associated with the Sb-based hybrid metal halide glasses allow for easier shaping and molding into complex or delicate structures. This flexibility can be particularly advantageous in applications requiring precision and customization, such as optical components and specialized glass products.

### 3.5. Other Hybrid Metal Halide Systems

**Figure 9:**
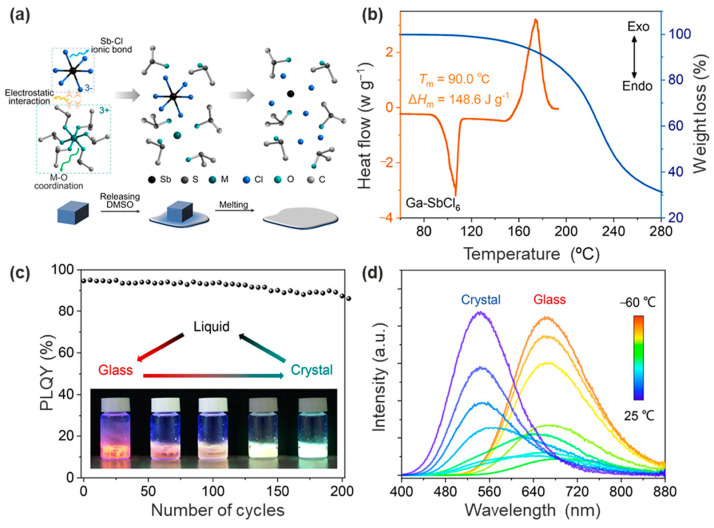
(**a**) Schematic diagram of glass formation for [M(DMSO)_6_][SbCl_6_] by DMSO release. (**b**) TGA (blue) and DSC (orange) of Ga-SbCl_6_. (**c**) PLQY of Ga-SbCl_6_ crystals during 200 cycles of melting and quenching. (**d**) Variable-temperature PL spectra of Ga-SbCl_6_ glass and crystals. Reproduced with permission from ref. [92]. Copyright 2023, American Chemical Society.

Although Pb-, Sn-, Mn-, and Sb-based hybrid metal halides are the most popular candidates for glass studies [94], recent studies have unveiled that for other glass systems with uncommon metal ions, their glass formation ability and glass properties are also intriguing. For example, the use of bulky organic cations, especially chiral molecules, helps to weaken the ionic bonding and makes it easier to get the material *T_m_* below 200 °C, which facilitates the preparation of glasses by melt quenching methods [22]. However, a weakening of the electrostatic interactions between the ions will inevitably lead to a decrease in the stability of the hybrid metal halides. To this end, Yao and coworkers built a series of 0D binary octahedral metal halides [M(DMSO)_6_][SbCl_6_] (M = Ga, Al, Y or Zr, etc.; DMSO = dimethyl sulfoxide) (denoted as M-SbCl_6_) to further reduce *T_m_* to below 100 °C (Figure 9a,b) [92]. Crystal structure analysis showed that the [M(DMSO)_6_]^3+^ and [SbCl_6_]^3−^ octahedra are packed in the trigonal crystal structure ($R\bar{3}$ space group) by their electrostatic interactions. The low *T_m_* of M-SbCl_6_ was attributed to the weak interaction of Ga-O coordination, which facilitated the release of DMSO from [M(DMSO)_6_]^3+^ and consequently dissolved [SbCl_6_]^3−^ octahedra. At lower temperatures (−60~−15 °C), [SbCl_6_]^3−^ exhibits an octahedral structure due to the strong Sb-Cl ionic bonding, while Ga^3+^ and DMSO are distributed around it in a disordered manner, which makes Ga-SbCl_6_ a glassy phase. When the temperature was raised to room temperature, the mobility of DMSO changed, leading to the transformation of Ga-SbCl_6_ glass into [Ga(DMSO)_6_][SbCl_6_] crystals, which the researchers speculated to have a *T_g_* of less than 0 °C. The optical study showed that the [Ga(DMSO)_6_][SbCl_6_] crystals exhibited bright photoluminescence with a nearly 100% quantum yield, attributed to the radiative recombination of self-trapped excitons formed by the Jahn−Teller-like distortions in the excited state of [SbCl_6_]^3−^ octahedra [95]. Since the [SbCl_6_]^3−^ center remains stably present in both glass and crystal, the PLQY of both phases can remain extremely high (>90%) after hundreds of phase transitions (Figure 9c). Notably, during the glass-crystal transition, the internal arrangement of the molecules was changed from disordered to ordered, and the energy of the self-trapped exciton was elevated, resulting in a change in photoluminescence from red to green (Figure 9d). This study provides important information for a deeper understanding of the mechanism and optical properties of the glass-crystal transition.

Except for Ga-based hybrid metal halide glasses, other metal halide systems containing Ge and Cu have also been investigated. As a group IVB element, Ge has similarly prompted further investigations into its physical and chemical properties by Mitzi’s group [38]. The Ge-based 2D metal halide (C_4_H_9_NH_3_)_2_GeI_4_ exhibited a larger, distorted octahedral structure and pronounced lone-pair stereoactivity compared to similarly structured Sn and Pb systems. The experimental results showed that the (C_4_H_9_NH_3_)_2_GeI_4_ sample, heated in argon, exhibited a lower melt temperature (i.e., 222 °C) than that of Sn (256 °C) and Pb (285 °C) systems with poorer melt stability. For Cu-based hybrid metal halide glasses, the construction of [Cu_4_I_4_L_4_] nanoclusters in cubic configuration is an effective strategy to achieve the stability of the melt even at high temperatures [96]. Compared with N-containing organic ligands, Bakr and coworkers reported the use of [Cu_4_I_4_L_4_] nanoclusters with P-containing organic ligands to enhance stability [97]. P-organic ligand-containing [Cu_4_I_4_L_4_] nanocluster glasses were successfully prepared in an air environment using the melt rapid cooling technique, possessing reversible transition properties between glassy, crystalline, and liquid phases. Among them, the weight loss of [Cu_4_I_4_(PPh_2_Et)_4_] nanocluster glass is only 0.1% at *T_m_*, demonstrating the high stability of its melt. After a rapid cooling treatment in an ice-water bath, the glassy material exhibited a light transmittance of more than 90%, which was attributed to the relatively weak van der Waals interactions between the nanoclusters and the strong CH–π interactions. It is also found that the number of alkyl chains in the P-based organic ligands severely affects the thermodynamic properties of the [Cu_4_I_4_L_4_] nanocluster glasses: the higher the number of alkyl chains, the better the flexibility and the weaker the rigidity of the P-based organic ligands, and the lower the *T_m_* and *T_g_* of the corresponding nanocluster glasses. This suggests that the more flexible organic ligands have a certain promotion effect on the formation of Cu-based nanocluster glasses.

**Figure 10:**
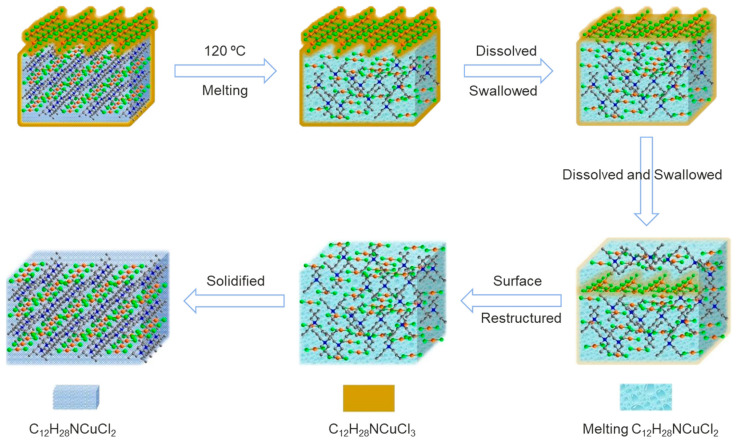
Schematic diagram of the valence transition mechanism of Cu-based hybrid metal halides. Reproduced with permission from ref. [16]. Copyright 2023, American Chemical Society.

To avoid potential oxidizing reactions for the Cu^+^ in the Cu-based hybrid metal halides, reducing their *T_m_* is very necessary for the preparation of Cu-based hybrid metal halide glasses. To this end, Jiang and coworkers reported two Cu(I)-based metal halides, C_12_H_28_NCuCl_2_ and C_20_H_48_N_2_Cu_4_Cl_6_, which have low *T_m_* of 86 and 122 °C, respectively [16]. Note that C_12_H_28_NCuCl_2_ was recorded as the most meltable metal halide among all metal halides at present, as far as we know. During the cooling of the melt, C_12_H_28_NCuCl_2_ exhibits a unique valence transition in the Cu^+^ ion, undergoing a solid–liquid–solid phase transition (Figure 10). In this process, the solid–liquid phase shows properties of fluorescence quenching and emits efficient green light. While melting, the external C_12_H_28_NCuCl_2_ oxidizes into C_12_H_28_NCuCl_3_, encasing the internal, unoxidized C_12_H_28_NCuCl_2_. As temperatures increase, this internal compound melts, releasing residual H_3_PO_2,_ which then reduces the C_12_H_28_NCuCl_3_ back to C_12_H_28_NCuCl_2_ with the Cu^+^ ion. This reversible self-restorative process is particularly valuable, providing insights for developing glass materials from Cu-based metal halides.

### 3.6. Crystalline–Amorphous Heterojunction

**Figure 11:**
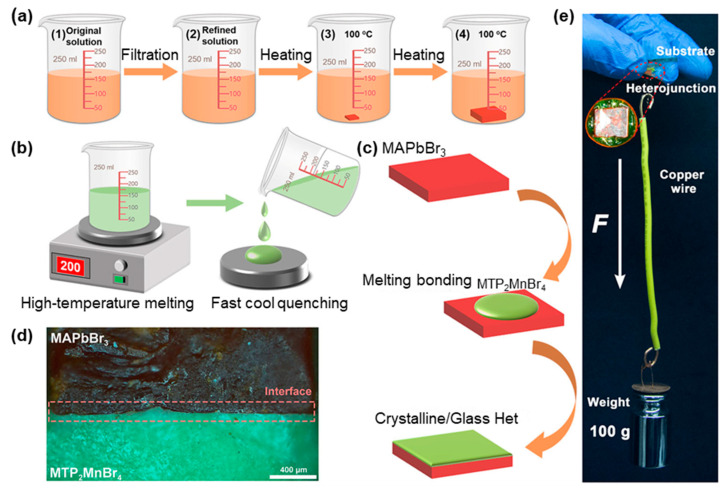
(**a**–**c**) Schematic illustrations of the preparation of MAPbBr_3_ single crystals, glass, and crystalline–amorphous phase heterojunction, respectively. (**d**) Cross-section of the crystalline–amorphous phase heterojunction. (**e**) Photograph showing tight bonding at the interface between crystalline and amorphous phases. Reproduced with permission from ref. [101]. Copyright 2023, Wiley-VCH GmbH.

The heterogeneous structure, comprising both crystalline and amorphous phases, exhibits heterogeneous interfaces with a distinctive atomic arrangement and a high concentration of active sites. On the one hand, compared to its pristine amorphous counterpart, this interface is believed to be conducive to facilitating efficient charge transfer and accelerating the kinetics of interfacial reactions [98]. Therefore, it has a wide range of potential applications, such as electrocatalysts [99] and photovoltaic cells [100]. On the other hand, in comparison to pristine crystalline, the interface can inhibit ion migration under an electric field and reduce noise interference in X-ray imaging [101,102]. To the best of our knowledge, the reported cases are still in the minority due to the difficulty of preparation. The first report on crystalline–amorphous heterojunction (i.e., MAPbBr_3_–MTP_2_MnBr_4_) for the improvement of X-ray imaging performance was proposed by Jin and coworkers [101]. The MAPbBr_3_–MTP_2_MnBr_4_ heterojunction was prepared by pouring the MTP_2_MnBr_4_ melt onto a prefabricated MAPbBr_3_ crystal with a flat surface (Figure 11a–c). When the MTP_2_MnBr_4_ cooled to form an amorphous phase glass, the melt gradually formed a new bond with the MAPbBr_3_ crystal, leading to the crystal–amorphous heterojunction with a flat cross-section and a tight bond (Figure 11d,e). Note that the high formation energy lattice defects at the interface increase the energy required for ion migration, leading to the difficulty of ion migration. As a result, the device exhibits excellent performance at a bias voltage of 200 V with a dark current as low as 0.12 nA.

## 4. Emerging Applications

With the development of theoretical and experimental studies on the thermodynamic and kinetic processes, the successful preparation of high-quality hybrid metal halide glasses has provided great opportunities for a variety of technological applications encompassing lighting, displays, information storage, and so forth. Owing to the merits of high X-ray attenuation coefficient, high light yield, and high optical transparency, high-quality hybrid metal halide glasses are particularly suitable for constructing scintillators for X-ray imaging.

### 4.1. Lighting and Displays

**Figure 12:**
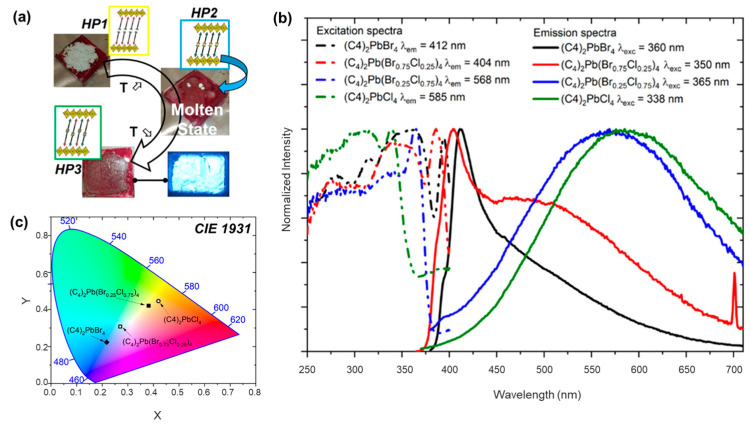
(**a**) Schematic of a solvent-free process (synthesis in the molten state). (**b**) PL and PLE spectra of (C4)_2_Pb(Br_1−x_Cl_x_)_4_. (**c**) The corresponding chromaticity diagram for (C4)_2_Pb(Br_1−x_Cl_x_)_4_ glasses. Reproduced with permission from ref. [17]. Copyright 2023, American Chemical Society.

Phosphor-converted LEDs (*pc*-LEDs) are a significant advancement in LED technology, offering efficient and versatile lighting solutions across various applications, from general lighting to displays [103]. *pc*-LED technology revolves around converting the light emitted by a blue or ultraviolet LED to different colors using luminescent materials such as rare-earth activated phosphors and semiconducting nanocrystals [104,105,106]. This conversion process allows for the creation of LEDs that can emit a broad spectrum of light, including white light, which is particularly useful in lighting applications [107,108,109]. Conversion efficiency and color tunability are two key indices for *pc*-LEDs. Due to their efficient light emission and high spectral tunability, hybrid metal halides are able to provide a wide range of color variations as phosphors in *pc*-LEDs [110,111]. In addition, the facile preparation for hybrid metal halide glasses provides convenience for their *pc*-LEDs applications. In a recent study, Mercier and coworkers reported a series of monolayered hybrid lead halides (HO_2_C(CH_2_)*_n_*_−1_NH_3_)_2_PbX_4_ (*n* = 4−6, X = Cl, Br) by varying chain lengths of organic cations through a molten method (Figure 12a) [17]. Notably, the research identified that these metal halides not only melt congruently at significantly lower temperatures compared to their traditional counterparts but also exhibit enhanced stability in the molten state. This behavior allows for the creation of thin films and new phases directly from the melt, bypassing the need for solvents. The ability to synthesize metal halides through this molten state method represents a major leap in material processing, offering a more environmentally friendly alternative to traditional practices. Moreover, the technique provides a pathway to manipulate the emission properties of these materials by adjusting the composition during the molten state, which is vital for tailoring the materials’ properties for specific applications, such as tunable light LEDs. For instance, the mixed anionic halides significantly alter luminescence colors due to the change of energy bands, spanning the spectral range from blue to yellow (Figure 12b,c). The implications of this research are profound, particularly for the advancement of solvent-free fabrication techniques that could lead to more sustainable industrial processes. Looking ahead, the study suggests potential areas for further research, including exploring the limits of congruent melting across different classes of metal halides and expanding the range of functional properties achievable through molten-state synthesis. Future investigations might also delve into the study of thermal regulation of the hybrid metal halide glasses as well as the scalability of this process and its integration into commercial manufacturing processes.

Generally, the photoluminescence quantum yield (PLQY) of the glass phase is expected to be lower than that of the crystalline counterpart, because the large number of defects within the highly disordered structures induces severe nonradiative recombination in the glass. Indeed, most of the metal halide glasses exhibit a PLQY lower than that of the crystals, presenting a challenge for their lighting applications [112,113]. However, recent studies have shown that although amorphous metal halides have a slightly lower emission intensity than crystals, they still have a relatively high PLQY. For example, Bakr and coworkers reported the synthesis of a series of cubic nanoclusters [Cu_4_I_4_(PR_3_)_4_] coordinated by a phosphine ligand (PR_3_) to form solid fusion-quenched glasses [97]. These nanoclusters were chosen due to their rich photophysical properties and potential for achieving reversible crystal–liquid–glass transitions. Through a meticulous process of melt-quenching in ambient conditions, the researchers successfully synthesized transparent glasses that retain the intricate structural integrity of the nanoclusters. One of these nanocluster glasses, designated as [Cu_4_I_4_(PPh_2_Et)_4_] glass, exhibited a PLQY greater than that of their corresponding crystals. The most notable finding of the study is the demonstration of over 90% transmittance across visible to near-infrared wavelengths, minimal self-absorption, and an exceptional photoluminescence quantum yield close to unity in the glass phase. This exceptional optical clarity and efficiency are attributed to the strong inter-nanocluster CH−π interactions that reduce structural vibrations, enhancing luminescence properties. The ability to maintain atomically precise structures within a non-crystalline matrix represents a substantial advancement in materials science, suggesting potential for a new class of materials that combine the processability of glasses with the desirable properties of crystalline materials. This breakthrough is particularly significant for *pc*-LEDs, where balanced optical properties are crucial.

Another prevalent issue encountered in the field of *pc*-LEDs is thermal management [114,115,116], which is a critical aspect in the design and operation of *pc*-LEDs due to its significant impact on the stability, efficiency, and lifespan of these devices [117,118]. To manage these thermal issues, as the resin materials exhibit low thermal conductivity (0.1~0.2 W m^−1^ K^−1^) [119,120], *pc*-LED designs often incorporate heat sinks, improved thermal interfaces, and sometimes active cooling mechanisms to dissipate heat more effectively. Advanced materials that can withstand higher temperatures without degradation are also being developed to enhance the thermal stability of *pc*-LEDs. In a typical study, Bennett and coworkers explored the melting behaviors of the manganese-based hybrid perovskites [TPrA][M(dca)_3_] (TPrA = tetrapropylammonium; M = Mn, Fe, Co; dca = dicyanamide), utilizing an innovative approach that combines experimental techniques with computational analysis to investigate their melting points and thermal behaviors [79]. The research revealed that these materials have lower melting points compared to traditional metal-organic frameworks, making them suitable for melt-quenching into glass. This process results in materials that retain the coordination between organic and inorganic components, presenting an intact network in the glass phase. The similar thermal conductivity (0.258~0.228 W m^−1^ K^−1^) of [TPrA][M(dca)_3_] indicates that the organic component plays a dominant role in the thermal conductivity properties of these materials. Although the values of thermal conductivity are lower than those previously reported for pure inorganic chalcogenides such as lead halide perovskites (0.4~0.5 W m^−1^ K^−1^) and cobalt-based perovskite (~1.3 W m^−1^ K^−1^), they are still higher than those of resin materials. The ability to create glasses from hybrid metal halides by melt-quenching opens up new avenues for the use of these materials in areas where glass-like properties are advantageous, and it also highlights the potential of these materials to bridge the gap between traditional crystalline and non-crystalline materials, offering a new toolkit and a solution for improving the thermal management of *pc*-LEDs.

### 4.2. Information Storage

**Figure 13:**
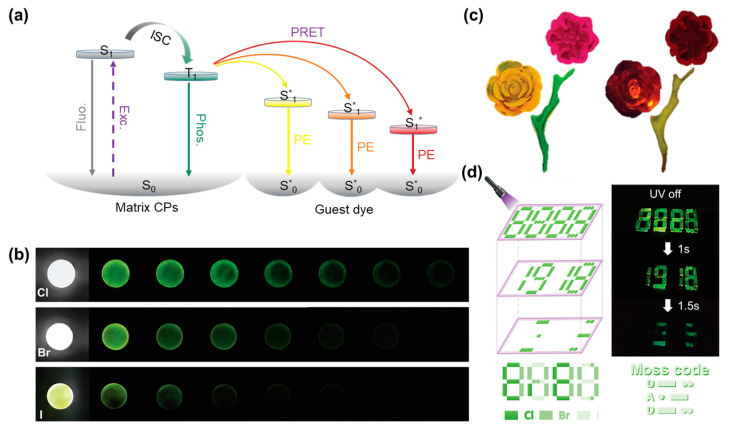
(**a**) The phosphorescence induced by energy transfer in ZnCl_2_(bIm)_2_ glasses. S_0_, S_1_, and T_1_ stand for the ground state, singlet excited state and triplet excited state of matrix, respectively. S*_0_ and S*_1_ stand for the ground state and singlet excited state of guest dye, respectively. (**b**) Halogen-dependent phosphorescence lifetime of ZnX_2_(bIm)_2_ glasses. (**c**) Photographs of jigsaws printed from ZnCl_2_(bIm)_2_ under UV on/off conditions, respectively. (**d**) Schematic and example of multiple-level information storage using ZnX_2_(bIm)_2_ glasses. Reproduced with permission from ref. [126]. Copyright 2024, Wiley-VCH GmbH.

The use of optical information storage in coding and anti-counterfeiting has grown significantly with advancements in technology, providing a reliable and secure method for protecting information and verifying authenticity in an increasingly digital world [121,122]. Optical storage can be engineered to include specific optical signatures or holographic watermarks that are extremely difficult to forge. Hybrid metal halide glasses with room-temperature phosphorescence have garnered attention due to their distinctive time-resolved emission properties [123,124,125]. In a recent study, Gong and coworkers reported a series of Zn-based metal-organic complex glasses that leverage the properties of coordination polymers combined with benzimidazole and various halides (Cl, Br, and I) to form glasses via melt-quenching [126]. Through the co-assembly of a long-lived hybrid glass donor and a dye acceptor in the glass matrix, the glasses show delayed sensitization of the acceptor’s single-linear state, resulting in delayed fluorescence (Figure 13a). The glasses display tunable room-temperature phosphorescence from 520 to 630 nm, with phosphorescence lifetimes varying significantly based on the halide used (Figure 13b). The researchers achieved dynamic control over these properties by manipulating the molecular rigidity and spin-orbital coupling effects, which are essential for extending the utility of these materials in display applications. Note that the ability to tune the spectral output and decay times of phosphorescence in these glassy materials marks a significant breakthrough. This control enables the precise design of materials for specific functions, such as multi-mode optical information storage and dynamic anti-counterfeiting systems (Figure 13c,d), which are increasingly vital in our digital age. It is worth noting that these hybrid glass materials can be processed into three-dimensional shapes, providing opportunities for the design of more complex anti-counterfeiting and advanced information storage applications in comparison to the flat patterns typically displayed by pure organic phosphors.

**Figure 14:**
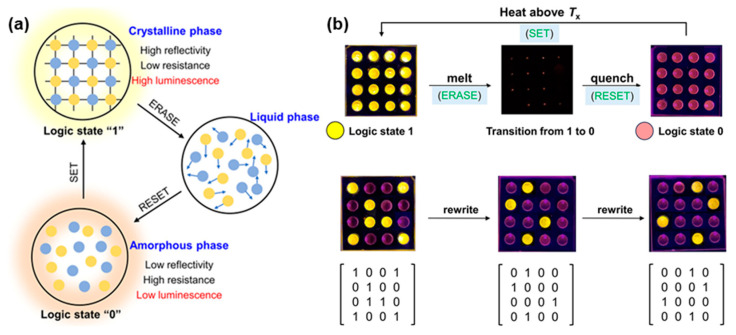
(**a**) Schematic of rewriteable data storage using photoluminescence switching during phase changes. (**b**) The demonstration of phase-change application using (*S*-2-HMM)_3_SbCl_6_ for thin film displays showing the color changes among different phases (**top panel**) and non-volatile memory and information storage (**bottom panel**). Reproduced with permission from ref. [24]. Copyright 2023, American Chemical Society.

The low melting point of hybrid metal halide glasses offers a good opportunity for the design of phase-change materials [17,35,92]. The strong property contrast between the different phases during the phase transition bodes well for the future of metal halides in fields such as information storage technology, non-volatile memory, and neurologically inspired computing [93,127,128]. Nevertheless, the restricted switching characteristics present a hindrance to the advancement and utilization of phase-change materials. Additionally, the design and control of the onset of the glassy phase, that is, the phase-transition temperature, is another challenge for phase-change materials that is determined to be a key parameter in determining the operating conditions and stability of phase-change materials [129]. In a recent report, Zhang and coworkers presented a kind of hybrid metal halide (*S*-2-HMM)_3_SbCl_6_ as a photoluminescent phase-change material [24]. The lower glass transition temperature (295 K) provides a foundation for the application of phase-change materials. The crystalline phase displays a near-unity photoluminescence with a quantum yield of 95%, which is attributed to the radiative recombination of self-trapped excitons in the excited state of [SbCl_6_]^3−^ octahedra. When the compounds undergo a stable melt process and become amorphous glass by melt-quenching, the glass phase exhibits an orange color with a very low quantum yield and maintains good transparency within the wavelength range from 400 to 800 nm (Figure 14a). The notable shift in photoluminescence occurs during the crystal-glass transition and demonstrates a pronounced photoluminescence switching capability. Interestingly, by heating, melting, and cooling different regions, transitions between different phases can be achieved to enable the writing and erasing of information (Figure 14b). The information can be readily discerned by monitoring the distinct luminescent states of these regions. The robust crystal-liquid-glass phase changes in hybrid metal halides and drastic photoluminescence switching open a new avenue to phase-change materials for further applications in remote information storage, sensing, and display [24].

### 4.3. X-ray Imaging

**Figure 15:**
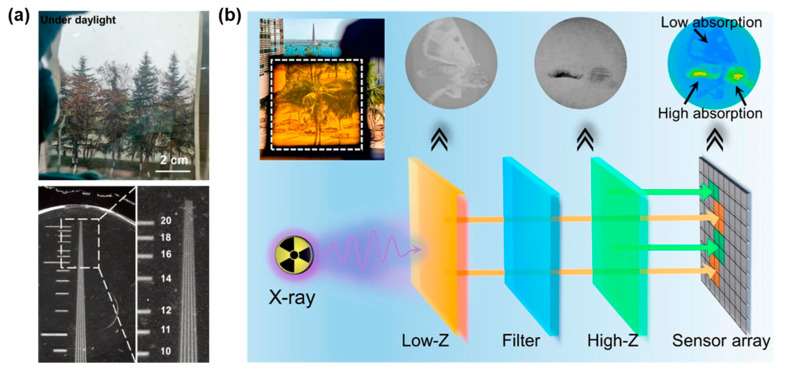
X-ray imaging applications induced by hybrid metal halide glasses. (**a**) A photograph of 10 cm × 10 cm (ETP)_2_SbCl_5_ wafers in daylight (**top panel**) and an image of the line pair card under X-ray using (ETP)_2_SbCl_5_ as a scintillator (**bottom panel**). Reproduced with permission from ref. [91]. Copyright 2023, Wiley-VCH GmbH. (**b**) Schematic of single-source dual-energy X-ray imaging. Inset: Photograph of the TFB sandwich structure scintillator under ambient light. Reproduced with permission from ref. [145]. Copyright 2023, American Chemical Society.

The ever-growing need to inspect matter with hyperfine structures requires a revolution in current X-ray imaging detectors, and the innovation of scintillators is revived with luminescent metal halides entering the scene [130,131,132,133]. Metal halides have garnered significant attention for their robust X-ray absorption and high optical output. However, despite their potential, metal halide scintillators are still far from reaching the commercial viability of established scintillators, primarily due to challenges in producing large-size crystals [133,134,135]. Although the nanocrystal-encapsulation technique has been developed to overcome the size issue, the problem still exists since nanocrystals embedded in polymers or glass are plagued by light tampering and reduced optical yield compared to single crystals [136,137,138,139,140,141,142]. Instead, hybrid metal halide glasses are then considered promising scintillating materials in terms of their high transmittance and flexible ductility [15,81,82,83,143,144]. For example, recently Liu and coworkers reported the high-quality glassy scintillator (ETP)_2_SbCl_5_, with a transmittance of 86% in the 450–800 nm range and a spatial resolution of up to 19 lp mm^−1^ (Figure 15a) [91].

In addition to the construction of single-layer scintillators, the highly transparent hybrid metal halide glasses are also suitable for the design of tandem scintillators for dual-energy X-ray imaging. For example, Mohammed and coworkers designed a top-filter-bottom (TFB) sandwich structure scintillator that simultaneously absorbs low- and high-energy X-ray photons within a single exposure (Figure 15b) [145]. This TFB structure efficiently converts absorbed X-rays into distinct emission colors, improving the resolution and material specificity of the imaging process. The TFB scintillator integrates organic and manganese-halide glasses, leveraging their properties to optimize X-ray photon conversion. Consequently, an exceptionally high spatial resolution (approximately 18 lp mm^−1^) was achieved in the X-ray images of this TFB stack, even exceeding the majority of previously reported single-layer scintillators based on organics and metal halides. This advancement in scintillator design represents a major leap forward in X-ray imaging technology. By enabling high-resolution, dual-energy imaging in a single exposure, the TFB scintillator structure offers a more detailed and nuanced view of the scanned materials or biological tissues. The success of this scintillator design opens up new avenues for the development of advanced imaging systems that can provide even more detailed insights into the structural and chemical properties of materials.

**Figure 16:**
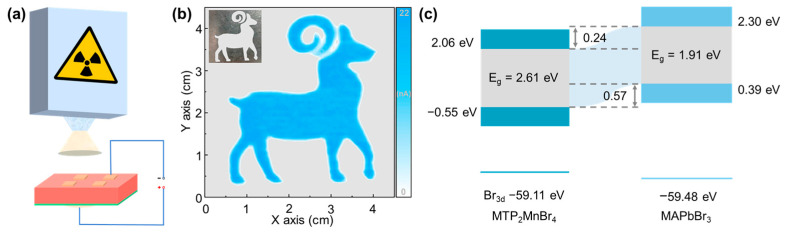
(**a**) Scheme of direct-type X-ray imaging. (**b**) Schematic diagram of energy band alignment for the MTP_2_MnBr_4_–MAPbBr_3_ heterojunction. (**c**) A pixelated X-ray imaging results of the “Sheep” metal plate. Reproduced with permission from ref. [101]. Copyright 2023, Wiley-VCH GmbH.

The fabrication of crystalline-amorphous heterojunction offers a promising solution to longstanding issues of noise caused by ion migration in direct-type X-ray detectors (Figure 16a). Through the design of heterojunctions, glassy metal halides have also been used for direct-type X-ray imaging. For example, Jin and coworkers reported a heterojunction comprising amorphous MTP_2_MnBr_4_ and crystalline MAPbBr_3_ (Figure 16b), which marks a substantial advancement in the field of X-ray detection, offering a method to enhance device performance without compromising detection capabilities [101]. Thanks to the high-quality heterojunction, the application to single-pixel X-ray imaging has been demonstrated by revealing the goat-shaped metal template (Figure 16c). The integration of crystalline and amorphous phases led to a significant improvement in X-ray detection sensitivity and noise reduction. It is worth mentioning that the heterojunctions showed excellent thermal stability and effectively suppressed ion migration, a common source of noise in metal halide-based detectors.

### 4.4. Optics and Sensing

**Figure 17:**
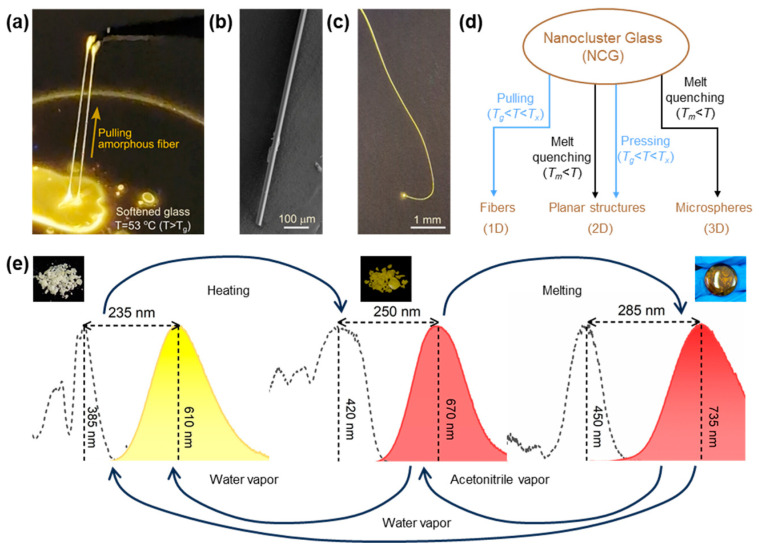
(**a**–**c**) Preparation, SEM image, and optical image of a hybrid glass fiber, respectively. (**d**) Schematic of the hybrid glasses with varied morphologies produced by different techniques; Reproduced with permission from ref. [97]. Copyright 2024, The authors, under the terms of the CC-BY 4.0 License. (**e**) Interconversion of the compounds (MTP)_6_SbBr_6_Sb_2_Br_9_·H_2_O (crystal 1), (MTP)_2_SbBr_5_ (crystal 2), and glassy (MTP)_2_SbBr_5_ (glass 3) and the corresponding change of spectra. Reproduced with permission from ref. [90]. Copyright 2022, Wiley-VCH GmbH.

Luminescent fibers can be engineered to detect and indicate various physical, chemical, and biological conditions, which can be crucial for diagnostics and monitoring in environments where electronics might fail or be impractical [146]. Glassy metal halides have unique low-temperature melting properties that offer significant advantages in material processing. Specifically, the ability of the material to transform into a liquid phase at relatively low temperatures allows it to be processed into a wide range of shapes and sizes by a variety of molding techniques, including blow molding, pressing, and stretching [147]. For example, Dong and coworkers fabricated transparent, flexible, low-loss glass hybrid metal halide glass fibers [Cu_4_I_4_(PR_3_)_4_] with diameters ranging from a few microns to several hundred microns and ultra-smooth surfaces (Figure 17a–c) [97]. The hybrid glass fibers were produced by pulling from the amorphous phase above its glass transition temperature. Note that, according to the available production techniques, various morphologies, such as planar structures and microspheres, can also be fabricated (Figure 17d). Typically, low propagation loss (~0.8 dB cm^−1^ at 567 nm) was achieved for the glass fiber of 200 μm in diameter, which is significantly lower compared to previously reported organic-inorganic hybrid glass waveguides [148,149,150]. Due to the low optical loss, the luminescent glass fibers exhibited strong light confinement and waveguiding effects, opening new avenues for advanced photonics applications due to negligible self-absorption and excellent homogeneity.

Metal halides are extremely susceptible to changes in environmental conditions, making them invaluable in optical sensors [151,152,153,154,155]. In a typical report, Xu and coworkers reported the interconversion of the (MTP)_6_SbBr_6_Sb_2_Br_9_·H_2_O crystals, (MTP)_2_SbBr_5_ crystals, and (MTP)_2_SbBr_5_ glasses (Figure 17e) [90]. The glassy phase undergoes a structural transformation upon contact with acetonitrile into (MTP)_2_SbBr_5_ crystals. Upon contact with water, both (MTP)_2_SbBr_5_ crystal glass and crystals undergo structural transformation into (MTP)_6_SbBr_6_Sb_2_Br_9_·H_2_O crystals. The structural transformation suggests that the solvent vapor has an induced recrystallization effect on the amorphous sample [156]. Accompanied by this structural transformation, the PL transforms from near-infrared luminescence at 735 nm in (MTP)_2_SbBr_5_ glasses to red emission at 670 nm in (MTP)_2_SbBr_5_ crystals and orange luminescence at 610 nm in (MTP)_6_SbBr_6_Sb_2_Br_9_·H_2_O crystals. The PLQY of the corresponding samples remained unchanged even after several transformation cycles by heating and cooling. Compared to the previously reported drawbacks of metal halides that are sensitive to solvents and prone to decomposition [157,158,159,160], the stability and sensitivity exhibited by the metal halides in the glassy phase lay the foundation for the application of metal halides in solvent detection [161,162].

## 5. Conclusions

This review article has comprehensively explored the burgeoning fields of glassy hybrid metal halides, underscoring significant strides made in synthetic strategies and elucidating the nuanced interplay of structural parameters that govern their formation and properties. The synthetic pathways, particularly melt-quenching and high-pressure compression, critically influence the amorphous characteristics of hybrid metal halides. The applications of glassy hybrid metal halides in technologies such as X-ray imaging and optoelectronics highlight their potential to renew these fields. Their unique properties, like highly tunable emission properties, make them suitable for lighting devices, reflecting their potential to contribute to sustainable technology solutions.

Despite the progress, several challenges and questions remain unaddressed, opening avenues for future research. Firstly, enhancing the understanding of the fundamental properties and behaviors of glassy hybrid metal halides through theoretical models and computational simulations is under way. Machine learning can help in predicting material behaviors and designing materials with specific desired properties before physical synthesis. Secondly, the stability and environmental impact of these glassy hybrid metal halides (especially for lead-based systems), particularly under operational conditions, require further investigation and assessment. Alternative compositions with non-toxic elements like tin and antimony should be explored more rigorously. Thirdly, developing glassy hybrid metal halides with tailored optical properties, such as specific refractive indices, transparency levels, and luminescent characteristics, is another promising direction. These materials could be optimized for use in optics and photonics, including lasers, lenses, and other light-manipulating components. In addition, research into cost-effective and scalable manufacturing processes for these materials would be crucial for their commercial viability, including the study of other fabrication techniques that can produce high-quality glassy materials at industrial scales. Last but not least, integrating insights from fields such as nanotechnology, materials science, optics, and environmental engineering could significantly advance the development of hybrid metal halide glasses. Standing at the frontier of materials science, we believe that glassy hybrid metal halides are continually offering promising prospects for both academic research and industrial application.

## Figures and Tables

**Table 1:** A summary of Pb-based hybrid metal halides.

Materials	Organic Cations	*T_x_* or *T_g_* (°C)	*T_m_* (°C)	*T_d_* (°C)	Ref.
(C4)_2_PbCl_4_	4-ammoniumbutyric acid	-	144.64	-	[17]
(C5)_2_PbCl_4_	5-aminovaleric acid	-	184.79	-	[17]
(C5)_2_PbBr_4_	5-aminovaleric acid	-	157.35	-	[17]
(C6)_2_PbCl_4_	6-aminocaproic acid	-	207.99	-	[17]
(C6)_2_PbBr_4_	6-aminocaproic acid	-	211.39	-	[17]
*Rac*-NPB	racemic 1-(1-naphthyl)ethylammonium	-	221	215	[22]
*S*-NPB	*S*-(−)-1-(1-naphthyl)ethylammonium	67 ^a^	173	205	[23]
(4-F-PEA)_2_PbI_4_	4-fluorophenethylammonium	244.3	258.9	-	[58]
(*β*-Me-PEA)_2_PbI_4_	*β*-methylphenethylammonium	170.7	207.0	-	[58]
(1-MeHa)_2_PbI_4_	1-methyl-hexylammonium	111.1	172.3	228	[63]
(2-F-PEA)_2_PbI_4_	2-fluorophenethylammonium	181.2	245.8	-	[64]
(4-MeO-PEA)_2_PbI_4_	4-methoxyphenethylammonium	214.2	247.1	-	[64]
(PEA)_2_PbI_4_	phenethylammonium	199.2	252.9	-	[64]
(3-F-PEA)_2_PbI_4_	3-fluorophen-ethylammonium	211.6	261.4	-	[64]
(DMIEA)_3_Pb_2_I_7_	*N*,*N*-dimethyl iodoethylammonium	-	173	235	[62]
(DMIPA)_4_Pb_3_I_10_	*N*,*N*-dimethyl iodopropylammonium	-	151	245	[62]
(DMBPA)_4_Pb_3_Br_10_	*N*,*N*-dimethyl bromopropylammonium	-	139	239	[62]

^a^ *T_g_* = 67 °C.

**Table 2:** A summary of Mn-based hybrid metal halides.

Materials	Organic Cations	*T_g_* (°C)	*T_m_* (°C)	Ref.
(HTPP)_2_MnBr_4_	hexyltriphenylphosphonium	38.6	169	[15]
(BuTP)_2_MnBr_4_	butyltriphenylphosphonium	46	~135	[35]
(1-EP)_2_MnBr_4_	1-ethylpyridine	-	~105	[35]
(1-BuP)_2_MnBr_4_	1-butylpyridine	-	~110	[35]
(BTP)_2_MnBr_4_	benzyltriphenylphosphonium	74	~220	[35]
(ETP)_2_MnCl_4_	ethyltriphenylphosphonium	-	204	[35]
(Bmmim)_2_MnCl_4_	1-butyl-2,3-dimethylimidazolium	-	50	[40]
(Bmmim)_2_MnBr_4_	1-butyl-2,3-dimethylimidazolium	-	60	[40]
(TPrA)[Mn(dca)_3_]	tetrapropylammonium	218	271	[79]
(BTA)_2_MnBr_4_	benzyltrimethylammonium		175	[80]
(ETP)_2_MnBr_4_	ethyltriphenylphosphonium	50	168	[81]
(BPTP)_2_MnBr_4_	3-bromopropyl)triphenylphosphonium	-	220	[82]
(HTP)_2_MnBr_4_	heptyl(triphenyl) phosphonium	-	165	[83]
(TP)_2_MnBr_4_	(triphenyl)phosphine	-	<220	[83]
(MTP)_2_MnBr_4_	methyl(triphenyl)phosphonium	55	<220	[83]
(PTP)_2_MnBr_4_	pentyl(triphenyl)phosphonium	-	<220	[83]
(TBuA)[Mn(dca)_3_]	tetrabutylammonium	33	185	[84]
(TPnA)[Mn(dca)_3_]	tetrapentylammonium	9	149	[84]

**Table 3:** A summary of Sb-based hybrid metal halides.

Materials	Organic Cations	*T_g_* (°C)	*T_m_* (°C)	*T_d_* (°C)	Ref.
(*S*-2-HMM)_3_SbCl_6_	*S*-2-(hydroxymethyl)morpholine	22	140	249	[24]
(*Rac*-2-HMM)_2_SbCl_5_	*rac*-2-(hydroxymethyl)morpholine	25	130	-	[24]
(Bmmim)_2_SbCl_5_	1-butyl-2,3-dimethylimidazolium	-	140	-	[40]
(TMPZ)SbI_5_	1,1,4,4 tetramethylpiperazinium	-	259	-	[89]
(*S*-MeTMPZ)SbI_5_	(*S*)-1,1,2,4,4-pentamethylpiperazinium	-	252	-	[89]
(MTP)_2_SbBr_5_	methyltriphenylphosphonium	65	178	-	[90]
(ETP)_2_SbCl_5_	ethyltriphenylphosphine	38	149	-	[91]
[Al(DMSO)_6_](SbCl_6_)	dimethyl sulfoxide	-	-	175	[92]
[Ga(DMSO)_6_](SbCl_6_)	dimethyl sulfoxide	~0	90	-	[92]
[Y(DMSO)_6_](SbCl_6_)	dimethyl sulfoxide	-	<100	-	[92]
[Zr(DMSO)_6_](SbCl_6_)	dimethyl sulfoxide	-	<100	-	[92]
